# Advances in the Structures, Pharmacological Activities, and Biosynthesis of Plant Diterpenoids

**DOI:** 10.4014/jmb.2402.02014

**Published:** 2024-06-17

**Authors:** Leilei Li, Jia Fu, Nan Liu

**Affiliations:** 1 School of Chinese Materia Medica, Tianjin University of Traditional Chinese Medicine, Tianjin 301617, P.R. China; 2 State Key Laboratory of Component-based Chinese Medicine, Tianjin University of Traditional Chinese Medicine, Tianjin 301617, P.R. China

**Keywords:** Plant diterpenoids, chemical structures, pharmacological activities, biosynthetic pathways, synthetic biology, heterologous production

## Abstract

More and more diterpenoids have attracted extensive attention due to the diverse chemical structures and excellent biological activities, and have been developed into clinical drugs or consumer products. The vast majority of diterpenoids are derived from plants. With the long-term development of plant medicinal materials, the natural resources of many plant diterpenoids are decreasing, and the biosynthetic mechanism of key active components has increasingly become a research hotspot. Using synthetic biology to engineer microorganisms into "cell factories" to produce the desired compounds is an essential means to solve these problems. In this review, we depict the plant-derived diterpenoids from chemical structure, biological activities, and biosynthetic pathways. We use representative plant diterpenes as examples to expound the research progress on their biosynthesis, and summarize the heterologous production of plant diterpenoids in microorganisms in recent years, hoping to lay the foundation for the development and application of plant diterpenoids in the future.

## Introduction

Diterpenes are widely distributed in plants, fungi, and marine organisms. The backbones of diterpenes are composed of four isoprene units that join head-to-tail. The complexity and diversity of diterpenoids make them rich in biological activities. Several plant diterpenes have been developed into clinical drugs to treat various diseases. For example, paclitaxel from *Taxus* is currently used for treating ovarian and breast cancer in clinical practice [1]. The tanshinones extracted from *Salvia miltiorrhiza*, a traditional Chinese medicine (TCM), are mainly used for treating cardiovascular diseases [2]. Ginkgolides, the active ingredients extracted from *Ginkgo biloba*, have medicinal effects in preventing and treating cardiovascular and cerebrovascular diseases [3]. In addition to medicinal values, diterpenoids have applications in industry and agriculture. For example, stevioside extracted from *Stevia* is 300 times sweeter than sucrose. It contains no calories and is regarded as the “third source of sugar in the world” [4]. Gibberellins are widely used to regulate the growth and development of plants in agriculture [5].

**Fig. 1:**
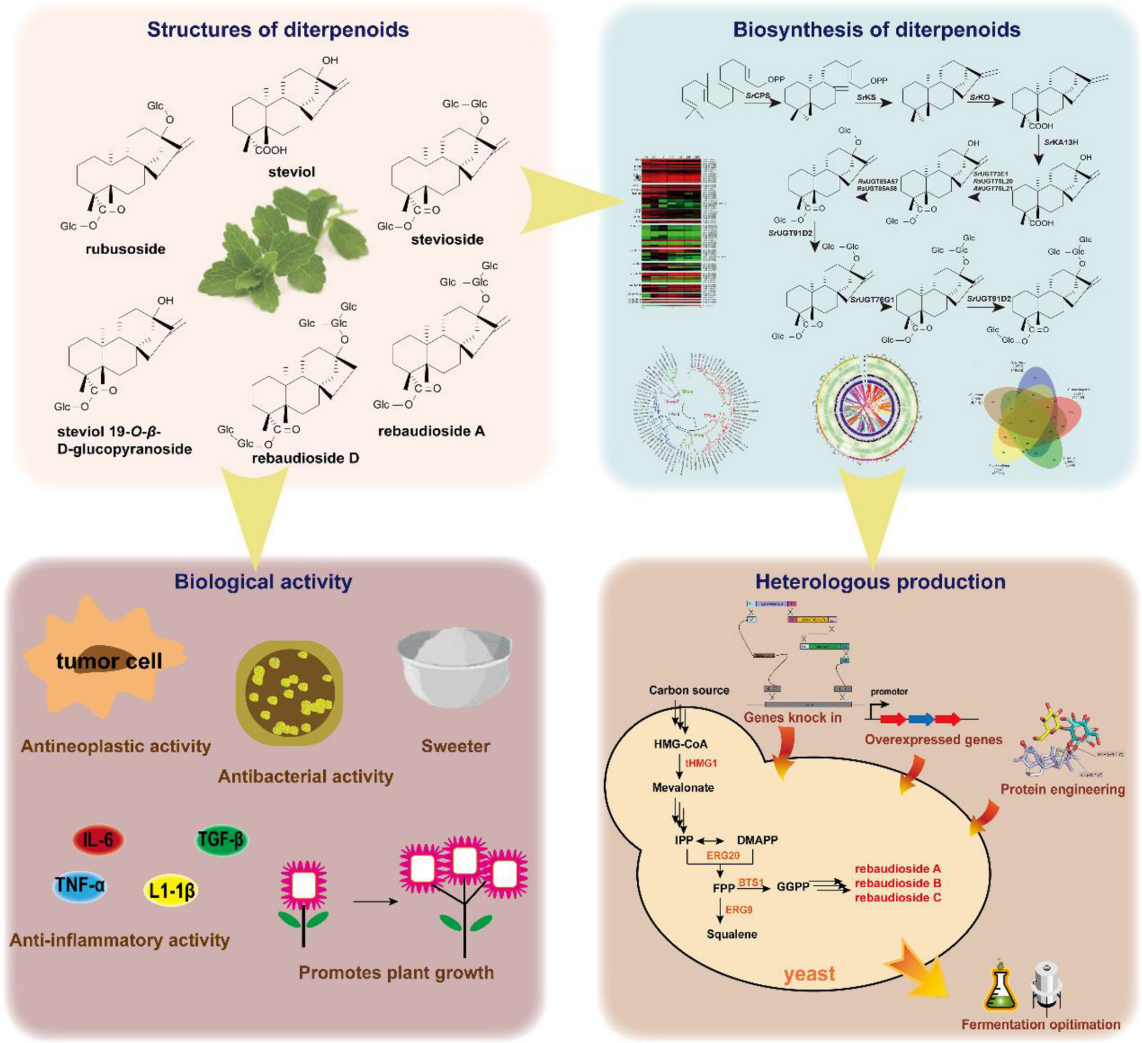
Comprehensive research strategies for plant diterpene resources, including their structures, biosynthetic pathway, heterologous production and biological activity (Adapted from [96, 126-130]).

Currently, various diterpenoids have been discovered in plants, showcasing higher diversity and content compared to animals and microorganisms. It seems that plant-derived diterpenes have higher purity and biological activity [6-8]. Therefore, the primary sources of diterpenoids rely on plant extraction. However, the low content of diterpenoids in plants, the long growth cycle of plants, and the complicated extraction and purification processes for diterpenes do not satisfy the requirements for green and sustainable diterpenoid production. In recent years, the chemical synthesis of many diterpenoids has been accomplished. However, due to the diverse structure of diterpenoids, the yield of total synthesis is often low and is not practical for industrial production. Therefore, exploring the biosynthetic pathways for plant diterpenoids and using the synthetic biology strategy to design and engineer microbial strains to yield plant diterpenoids has been recognized as a promising method to produce these crucial compounds (Fig. 1).

With the explosion of genome sequencing data and the increasing characterization of biosynthetic enzymes, the biosynthetic pathways of many diterpenoids have been elucidated, which lays the foundation for the heterologous production of diterpenoid products. Through metabolic engineering, synthetic biology, and fermentation engineering, researchers have developed environmentally friendly biosynthetic strategies and designed efficient cell factories to produce high-value diterpenoids (Fig. 1). Various microorganisms, including *Escherichia coli* and yeasts, have been exploited as chassis cells to produce diterpenoids. In recent years, a few plant diterpenoids or their structural analogs, such as taxadiene [9], tanshinone IIA [10], and steviol [11], have been produced in these microbial cell factories.

This review first describes the chemical structures and pharmacological activities of plant diterpenoids. Then we summarize the research progress of the biosynthetic pathways of a few diterpenes and the heterologous production of plant diterpenoids in microbial cell factories. We predict the prospects and development trends of synthetic biology in producing plant diterpenoids, aiming to provide more insights for future research and application of diterpenoids.

## Classification of Plant Diterpenoids

**Fig. 2:**
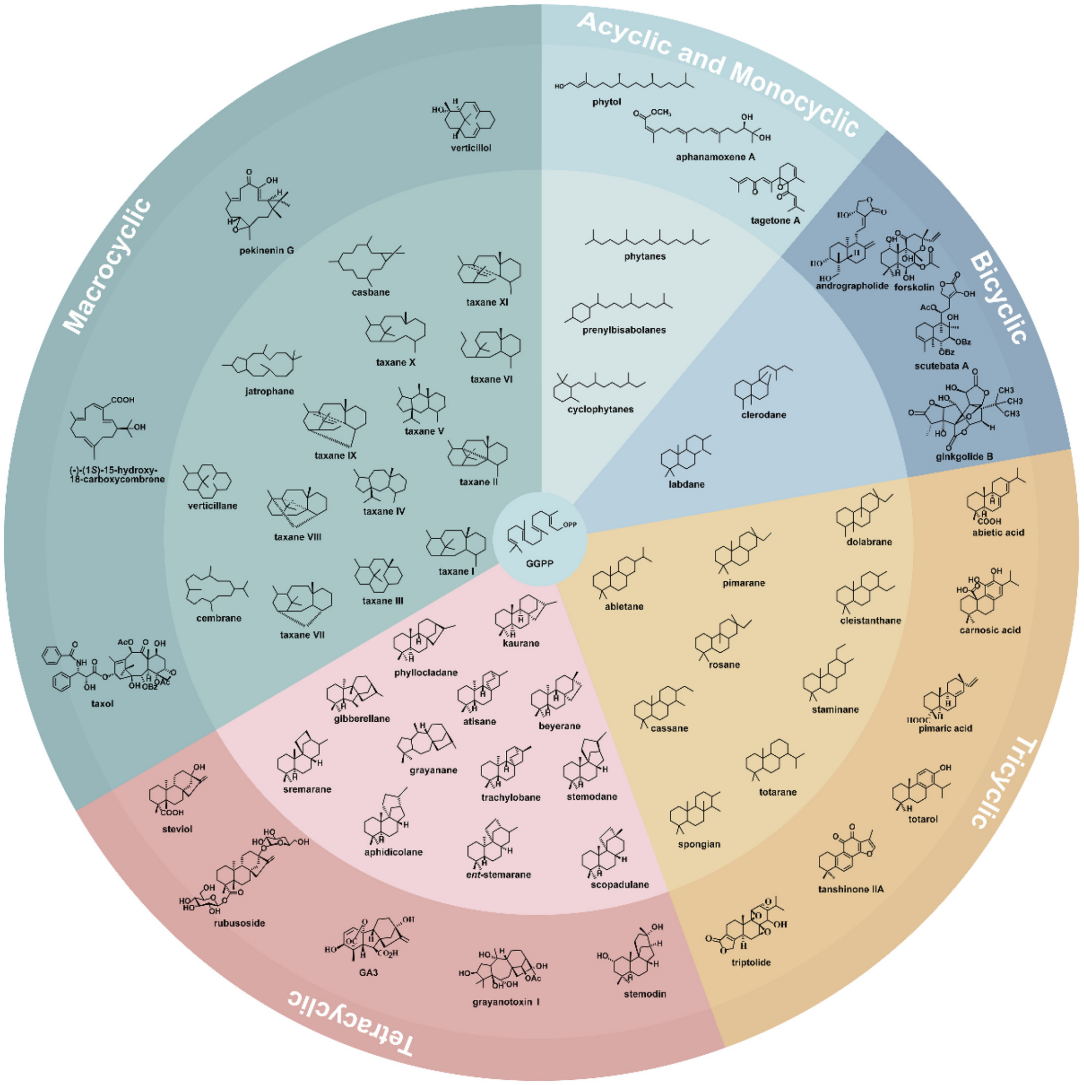
The different skeleton types of plant diterpenoids and the corresponding compounds. Acyclic and monocyclic diterpenoids (cyan), bicyclic diterpenoids (blue), tricyclic diterpenoids (yellow), tetracyclic diterpenoids (pink), and macrocyclic diterpenoids(green).

According to the number of skeleton rings, plant diterpenoids can be divided into acyclic, monocyclic, dicyclic, tricyclic, tetracyclic, and macrocyclic subfamilies. Besides sesquiterpenoids, diterpenoids have the most abundant structure types in terpenoids, which can be generated through the fracture, carbon bond shift, and skeleton rearrangement (Fig. 2). In this paper, we classify diterpenoids in plants based on the skeleton ring system.

### Acyclic and Monocyclic Diterpenoids

Acyclic and monocyclic diterpenoids are relatively rare in plants. Still, they have essential biological functions. Phytol is an acyclic diterpene commonly found in the leaves of green plants [12]. It acts as a side chain and binds to magnesium-containing porphyrins to form chlorophyll. At the same time, phytol is used as a precursor for the biosynthesis of vitamins E and K1 [13]. Recently, three new acyclic diterpenoids with strong anti-inflammatory activity, aphanamoxenes A, B, and C, were isolated from the seed of *Aphanamixis polystachya* [14] (Fig. 2). Ibrahim *et al*. identified two new monocyclic diterpenoids, tagetones A and B, from the flowers of *Tagetes minuta* [15] (Fig. 2).

### Bicyclic Diterpenoids

Bicyclic diterpenoids are mainly divided into the labdane and clerodane families. In addition, bicyclic diterpenoids can combine with other structure types to form new compounds, such as ginkgolides composed of sesquiterpene and diterpene structures [16].

**Labdanes.** Labdanes have naphthane as the parent nucleus and possess various structures formed by rearranging and C–C bond cleavage of the parent nucleus. They are divided into the labdane (*β*-CH_3_ at C-10 and *α*-H at C-5 and C-9) and *ent*-labdane (*α*-CH3 at C-10, *β*-H at C-5 and C-9) subfamilies (Fig. 2). Andrographolide and its derivatives isolated from *Andrographis paniculate* of the family Acanthaceae [17] and forskolin from *Coleus forskohlii* are two representative compounds in this class [18].

**Clerodanes.** Clerodanes have attracted attention because of their insect antifeedant activity. Most of them were found in Angiosperm. The clerodanes can be divided into two classes, clerodane and *ent*-clerodane [19]. Various clerodanes with antitumor activity have been found in *Scutellaria barbata* of the genus *Scutellaria* in Labiatae, such as scutebata A. It has cytotoxic activities against a variety of cancer cells, LoVo (colon cancer), MCF-7 (breast cancer), SMMC-7721 (hepatoma cancer), and HCT-116 (colon cancer) cells [20] (Fig. 2). Besides, six undescribed clerodanes were isolated from a traditional Uygur medicine, *Salvia deserta*, which can inhibit the secretion of cytokines TNF-α and IL-6 in macrophages RAW264.7, displaying immunosuppressive activity likely attributed to their possession of a terminal, *α*-unsaturated-*β*-lactone moiety [8, 21]. Furthermore, the clerodanes diterpenoids separated from levels of *Casearia coriacea* possess toxicity to malaria parasite with selectivity.

**Others.** Ginkgolides were isolated from *the Ginkgo biloba* of the Ginkgoaceae family. Ginkgolides include ginkgolides A, B, C, J, M, K, L, and bilobalide. Different from the other seven diterpenes, bilobalide is a sesquiterpene. Ginkgolides have a unique twelve carbons skeleton structure embedded with a tert-butyl group and six 5-membered cyclic, which include a spiro [4.4] nonane, tetrahydrofuran, and three lactonic rings [22](Fig. 2). Ginkgolides specifically inhibit the platelet-activating factor receptor (PAFR). It was found that the position and number of hydroxyl groups have a significant impact on the activity of ginkgolides, such as the hydroxyls of ginkgolides C and J are at the C-7 position, their activity are weaker than ginkgolide B whose hydroxyl is located on the C-1 position, and it has the highest activity [23].

### Tricyclic Diterpenoids

Tricyclic diterpenoids are abundant in nature and rich in biological activities, such as anti-tumor, anti-inflammatory, and antibacterial. Tricyclic diterpenoids mainly include abietane, pimarane, rosane, cassane, totarane, and spongian. They can be divided into two types: aromatic and non-aromatic, according to the structural characteristics of their C-ring skeleton.

**Abietanes.** In the 19th century, people found the first abietane diterpene from colophony, abietic acid. It has antitumor, antioxidant, and other activities [24]. Studies have shown that methyl abietate, abietinol, and abietinal can be obtained by modifying the carboxyl at the C-18 position through esterification, reduction, and oxidation. Through assessing their cytotoxicity, found that among them, methyl abietate showed the highest cytotoxicity, and abietinol presented weaker cytotoxicity [25]. Carnosic acid is also one of the representative tricyclic diterpenes, which was isolated from *Rosmarinus officinalis* of the family Lamiaceae. It is mainly used in the food processing industry (Fig. 2). This compound is recognized for its antioxidant, anti-inflammatory and cardiovascular protective effects [26-28]. Tanshinone IIA and tanshinone is an active component extracted from *S. miltiorrhiza*, a traditional Chinese medicine (Fig. 2). It can alleviate atherosclerosis by regulating macrophage polarization and has antitumor activity [29]. It was reported that introducing amino acids in the D-ring of tanshinone IIA could enhance its cytotoxicity against tumor cells [30].

**Pimaranes.** Like abietanes, pimaranes were also originally isolated from colophony. They can be divided into four types, namely, pimarane, isopimarane, *ent*-pimarane and *ent*-isopimarane. The difference between isopimarane and pimarane is in the configuration of the methyl group at C-17. Isopimarane has *α*-CH3 while pimarane has *β*-CH_3_. Pimaric acid is the first pimarane diterpene isolated from *Pinus sylvestris* of the genus *Pinus* in Pinaceae in 1983 (Fig. 2). Icacinol, a pimarane-type diterpene, was isolated from the tubers of *Icacina oliviformis* [31]. Four highly oxidized pimarane diterpeniods isolated from the rhizomes of *Kaempferia takensis* can inhibit the generation of nitric oxide (NO) in RAW264.7 macrophage cells and inhibiting NF-κB production in HaCat human skin cells [32].

**Others.** In addition to the two tricyclic diterpenes described above, there are several other types, such as rosane, cassane, totarane, and spongian. Totarol is a representative totarane diterpene isolated from the duramen of *Podocarpus macrophyllus* (Fig. 2) [33]. At present, many daily chemical products and cosmetics contain totarol. Triptolide is mainly extracted from *Tripterygium wilfordii*, its potency is 100-200 times higher than that of tripterygium glycosides, and its therapeutic window is narrow. Triptolide is currently the main component of the quality control specification of *T. wilfordii* preparations. (Fig. 2).

### Tetracyclic Diterpenoids

Tetracyclic diterpenoids can be classified into two types. The tetracyclic diterpenes with a C-8 bridging ring include kaurane, *ent*-gibberellin, phyllocladane, atisane, beyerene, and grayanane. Their biosynthetic intermediate is *ent*-CPP. The tetracyclic diterpenes with C-9 bridging ring include aphidicolane, stemodane, stamarane, *ent*-stamarane, and scopadulane, among which aphidicolane was only found in fungi, and the intermediate is *syn*-CPP.

**Kauranes.** Kaurene diterpenoids were initially discovered from the essential oil of the leaves of *Agathis australis*, a plant of the Araucariaceae family from New Zealand. More than 1000 kauranes have been identified [34], and most of them are from plants of the *Isodon* and Asteraceae families [35]. Steviol glycosides (STE-G) are representative kauranes isolated from *Stevia rebaudiana*. They are currently used as food sweeteners. Various STE-G can be formed by substituting the C-13 hydroxyl or C-19 carboxyl of steviol with different types and numbers of glycosyl groups. Rubusoside possesses one molecule of glucose at the C-13 and C-19 positions of steviol, respectively. Stevioside contains additional glucose at the C-13 position of rubusoside. Continuing to add glucose by the glycosyltransferase gives rebaudioside A, D, and M (Fig. 2).

**Gibberellins**. Gibberellins (GAs) are a family of important hormones that control plant growth and development. GAs share the gibberellane backbone and differ by changing the number and position of double bonds and hydroxyl groups. GAs were initially isolated from *Gibberella fujikuroi*, a rice pathogen, and were later found to exist in the plant and bacteria kingdoms. More than 130 GAs have been found [36]. According to the number of carbon atoms, GAs can be divided into 20C *ent*-gibberellane and 19C *ent*-gibberellane, and the latter is relatively more varied. GA3, GA7, and GA30, which possess similar structures to their A-ring, showed better activity. Because the content of GA3 in plants is too low to extract, it can be achieved by using a synthetic biology strategy, for example, Kildegaard *et al*. reconstructed the biosynthetic pathway of gibberellins in *Yarrowia lipolytica* to produce GA3 and GA4, successfully [37, 38] (Fig. 2).

**Atisanes.** Atisane diterpenoids mainly exist in nature as *ent*-atisanes. Nearly half of the atisanes were isolated from *Euphorbia*. Other genera that contain atisanes include *Isodon*, *Xylopia*, and *Spiraea*. Drummond *et al*. first isolated atisanes from the tropical tree *Erythroxylon monogynum* in 1965 [39]. Oxidative modification of these compounds often occurs at the C-3, C-16, and C-17 positions of the parent nucleus (Fig. 2). Wang *et al*. discovered two novel *ent*-atisane ebractenone A and ebractenone B, which possess the unusual 2-oxopropyl moiety, from the roots of *Euphorbia ebracteolata* [40].

**Others.** Phyllocladanes are rare in nature, and their structures are similar to kauranes. Calliterpenone and its analogs isolated from *Callicarpa macrophylla* have the activity of promoting plant growth [41]. Grayananes are derived from kaurene skeleton rearrangement in nature and are found only in Ericaceae plants [42]. These compounds share a unique 5/7/6/5 tetracyclic structure. Sun *et al*. isolated 24 grayanane diterpenoids from *Rhododendron auriculatum*, six of which exhibited significant analgesic effects at 5.0 mg/Kg [43]. Stemodin and stemodinone are the first reported stemodanes derived from *Stemodia maritima* of the genus *Stemodia* in the family Scrophulariaceae [44] (Fig. 2). Two years later, another compound with a new configuration, stemarin, was isolated from *S. maritima* [45].

### Macrocyclic Diterpenoids

**Taxanes.** At present, more than 500 taxanes have been found. They can be divided into four subfamilies, *i.e.*, taxanes, secotaxanes, abeotaxanes, and cyclotaxanes [46] (Fig. 2). Taxanes diterpenoids were mainly isolated from the *Taxus* of the Taxaceae family. Paclitaxel, one of the most famous anticancer drugs, was initially discovered in the screening of antitumor drugs, and its structure was first reported in 1971 [47]. In 1994, the total synthesis of paclitaxel was reported. However, because of the complexity and the high cost of total synthesis, it is unrealistic to produce paclitaxel by this method. And semi-synthesis and microbial fermentation are the most promising alternative methods [48].

Due to the side effects and poor water solubility of paclitaxel, plenty of research has been focused on its structure modification in recent years. Bouchet *et al*. modified the benzoylphenyl and acetyl groups of paclitaxel to alkoxy and hydroxyl groups, respectively, to afford docetaxel. Docetaxel binds to tubulin with twice the affinity of paclitaxel because of its smaller space steric hindrance and stronger hydrophilicity brought by the polar groups [49]. Singer *et al*. found that adding hydrophilic and non-pharmacological groups to paclitaxel can improve the antitumor efficiency and reduce the side effects, as exemplified by xyotax [50].

**Others**. Macrocyclic diterpenes are a class of compounds whose molecular skeleton consists of four isoprene units and has a ring structure of more than six members. Their structures are complex, and generally have variety of functional groups, with unique biological activities and pharmacological effects, such as cytotoxicity, anti-tumor, antiviral and anti-inflammatory activities. These compounds are mainly found in plants of Euphorbiaceae and Thymelaeceae [7]. Such as cembranes, tigliane, casbanes, and jatrophanes, which have become the research hotspot due to their different skeletal structures and extensive biological activities (Fig. 2). For example, the ingenol extracted from *Euphorbia kansui* or *Leptochloa chinensis* has significant anti-tumor and antiviral activity. Prostratin, a macrocyclic diterpenoid isolated from small shrubs of New Zealand, can inhibit the HIV-1 infection and reduce its incubation period [51].

Cembranes can be divided into isopropyl, five-, six-, seven-, and eight-membered lactone rings, ring-opening, reduced-carbon type, etc. A new cembrane diterpene, named (-)-(1S)-15-hydroxy-18-carboxycembrene, was isolated from the roots of *Euphorbia pekinensis* Rupr, which had inhibitory effects on five tumor cells, including Hela, PC-3, HT1080, A375-S2 and MDA231 [52]. Pekinenin G is isolated from *Euphorbia*, which belongs to casbanes. It showed different cytotoxicity against four kinds of tumor cells, including BGC-823, HT-29, MCF-7, and A549 [53]. Jatrophanes have a bicyclic 15-carbon skeleton and are a new class of P-glycoprotein inhibitors [54]. Verticillol, the first isolated verticillane compound (Fig. 2), was isolated from *Sciadopitys verticillate* in 1962. It is mainly distributed in *Taxu* and is considered to be the precursor of taxanes [55].

## Pharmacological Activities of Plant Diterpenoids

Diterpenoids have a wide range of biological activities due to their complex and diverse structure. They are commonly used to treat cardiovascular diseases, hypertension[56], and diabetes [57, 58]. These compounds also have antineoplastic, anti-inflammatory, antibacterial, antiviral [59], antioxidative [60], and antimalarial activities [61]. Herein, our paper systematically summarizes the pharmacological studies on diterpenoids.

### Antineoplastic Activity

The antitumor effect is one of the most interesting pharmacological activities of diterpenes. Paclitaxel, triptolide, and *ent*-kaurene are star compounds for treating cancer. Paclitaxel has a variety of antineoplastic activities, especially for ovarian cancer, endometrial carcinoma, and breast cancer, which have a high incidence. It has been reported that paclitaxel can exert antitumor effects by promoting tubulin polymerization and blocking the progression of mitosis. At the same time, it can also promote the proliferation and differentiation of T-lymphocyte and increase the number of nature killers [1].

It is well known that non-small cell lung cancer (NSCLC) is one of the most severe diseases, it is characterized by a high incidence of metastasis and poor survival, and epithelial-to-mesenchymal transition (EMT) is a major factor inducing tumor metastasis. Deng *et al*. found that triptolide could inhibit the EMT of NCL-H1299 cells in a concentration-dependent manner and suppress *β*-catenin expression in NCL-H1299 and NCL-H460 cells [62].

In recent years, *ent*-kaurane has also been shown to have high anticancer activity. For example, oridonin, a natural compound isolated from the genus *Isodon* in the Labiatae, is currently in phase I clinical trial in China. This compound has anticancer effects on various human cell lines, such as large intestine cancer, acute myeloid leukemia, and BxPC-3 [63]. Overall, diterpenoids offer diverse avenues for cancer treatment, for example, they can inhibit the growth and migration of cancer cells by suppressing the AKT signaling of the key proteins implicated in diseases [64, 65], or controlling cell cycle to induce senescence and death of cancer cells [65]. Furthermore, altering the structure of diterpenoids enables the control of toxicity selectivity, premyrsinanes isolated from *Euphorbia gedrosiaca* show safety against breast cancer cells [66].

### Anti-Inflammatory Activity

The pathogenesis of numerous diseases is closely linked to inflammation. Diterpenes exhibit significant anti-inflammatory properties, suggesting their potential therapeutic efficacy in a range of conditions including neuroinflammatory diseases, atherosclerosis, liver damage, and colitis. The inflammatory response induced by cytokines and chemokines can cause various inflammatory diseases, and anti-inflammatory drugs are an important therapeutic strategy. Tanshinone IIA and cryptotanshinone have significant anti-inflammation activities. The research showed that tanshinone IIA plays an anti-inflammatory role by inhibiting the expression of inflammatory cytokines and TLR2, NF-kappa B, and ICAM-1 [67].

(1*R*,2*R*,4a*S*,5*R*)-1-(hydroxymethyl)-1,4a-dimethyl-6-methylene-5-(2-(4-nitrophenoxy) ethyl) decahydronaphthalen-2-ol is a derivative of andrographolide. Wang *et al*. examined its inhibitory activity for LPS-induced NO production in the RAW264.7 macrophage. It was shown that its IC_50_ value (3.38 ± 1.03 μM) was higher than that of andrographolide (8.81 ± 1.03 μM). They found that andrographolide and its derivatives exert anti-inflammatory effects by down-regulating the expression of COX-2, iNOS, and NF-κB and inhibiting the signal pathway [68].

Wang *et al*. isolated twelve new diterpenoids from the leaves of *Euphorbia lathylris* and verified their anti-inflammatory effects. They found that euplarisan A showed anti-inflammatory effects with an IC_50_ value of 7.50 ± 1.45 μM. It could inhibit the expression of inflammatory cytokines in a dose-dependent manner, including iNOS, COX-2, and p-IκBα [69].

### Antibacterial Activity

Some diterpenoids have a broad antimicrobial spectrum, low toxicity, and high antibacterial activity. For instance, *Streptococcus mutans* has been recognized as the most important oral cariogenic pathogen. It was reported that rubusoside (50 mM) showed the 97% inhibitory activity against the mutansucrase in *S. mutans* with 500 mM sucrose. Therefore, it has particular research value and application prospects in preventing and treating caries [70].

Siddique *et al*. isolated a new labdane diterpene, named (*E*)-8(17), 12-labdadiene-15, 16-dial, from *Zingiber montanum*, it has potential antibacterial activity against a series of multi-drug and methicillin-resistant *Staphylococcus aureus* (MRSA) with minimum inhibitory concentrations (MIC) of 32–128 μg/ml. It has exomethylene at C-8, an olefine at C-12, and two aldehyde groups at C-16 and C-17. These are the reasons for its significant antibacterial activity against MRSA strains [71].

### Others

In addition to the above common activities, diterpenoids have numerous other biological activities. Such as andrographolide, 14-dehydroxyandrographolide-12-sulfonic acid sodium salt, and 14-*α*-lipoyl andrographolide have been found to inhibit several influenza viruses, including H5N1, H1N1, and H9N2. Among them, 14-*α*-lipoyl andrographolide inhibited the adsorption of the virus to erythrocytes. This suggested that the compound can interfere with viral hemagglutinin [72]. Andrographolide can also promote the regeneration of the skeletal muscle of mice. The experimental results show that it might up-regulate the transcription of myogenic differentiation genes to facilitate the differentiation and fusion of muscle cells by enhancing the methylation of histone H3K4, thereby promoting the regeneration of skeletal muscle [73].

Due to its high sweetness and low calorie, rubusoside is used as a sweetener. It also can combine with resveratrol, curcumin, and other anticancer drugs to increase solubility, such as by 33-fold of resveratrol and 60-fold of curcumin [74, 75].

GAs is a large group of endogenous hormones that can regulate plant vegetative and reproductive growth. They play important roles in promoting seed germination, root growth, stem and leaf growth, flowering, and fruit ripening [76]. Many diterpenoids showed noteworthy neuroprotective effects in cell injury models induced by H_2_O_2_ and MPP^+^ [77], they can activate the autophagia and trigger the parkin/IKK/p65 pathway to increase the OPA1. They can also show a neuroprotective effect by inhibiting the COX-2 pathway and MAPK pathway [78].

## Studies on the Biosynthesis of Plant Diterpenoids

**Fig. 3:**
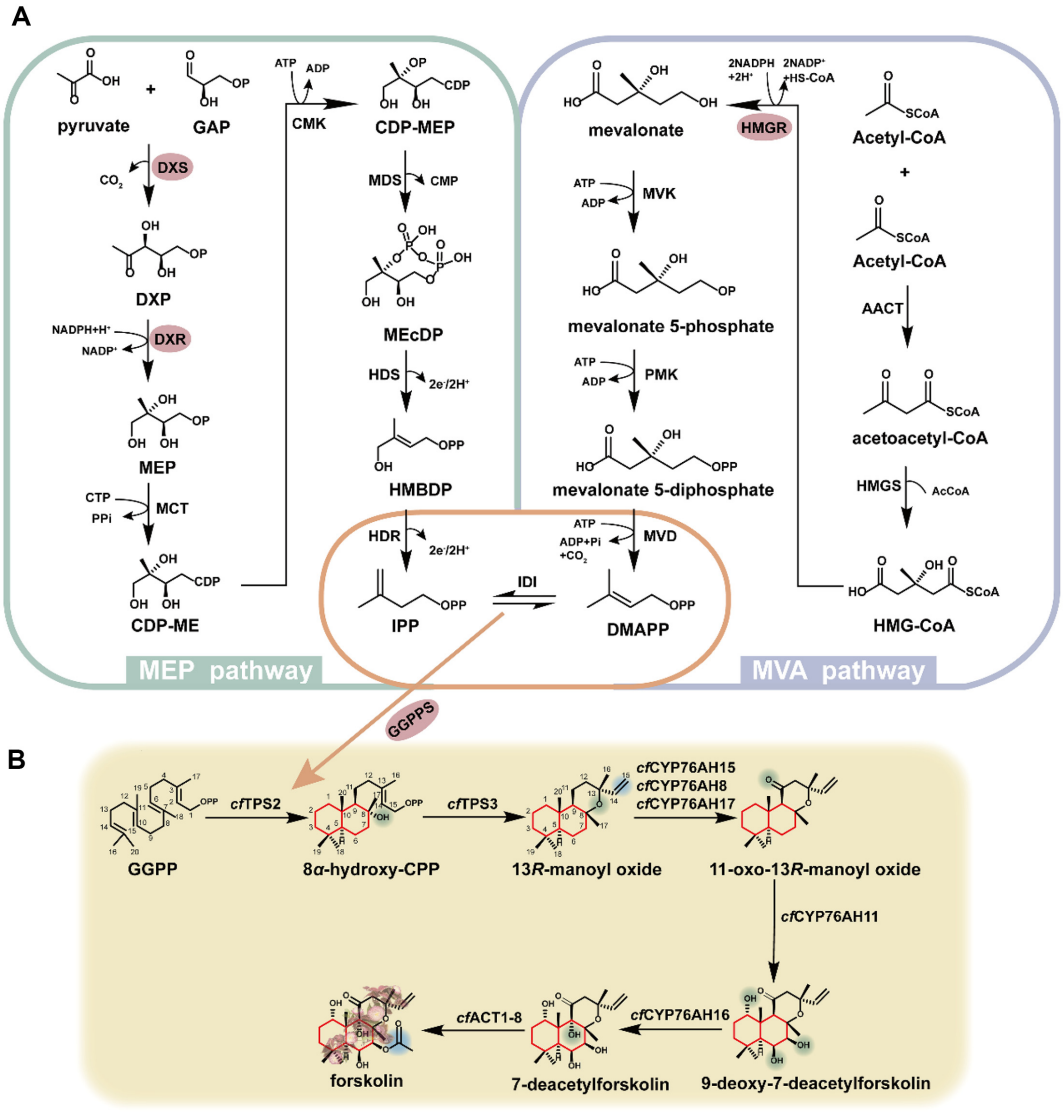
(A) Biosynthesis of the IPP and DMAPP via the MVA pathway (purple) and MEP pathway (green). Enzyme abbreviations are as follows; AACT, acetoactl-CoA thiolase. HMGS, hydroxymethylglutaryl-CoA synthase. HMGR, hydroxymethylglutaryl-CoA reductase. MVK, mevalonate kinase. PMK, phosphmevalonate kinase. MVD, mevalonate 5- phosphate decarboxylase. IDI, isopentenyl diphosphate isomerase. DXS, 1-deoxy-D-xylulose 5-phosphate synthase. DXR, 1- deoxy-D-xylulose 5-phosphate reductoisomerase. MCT, 2C-methyl-D-erythritol 4-phosphate cytidyltransferase. CMK, 4- (cytidine 5’-diphospho)-2C-methyl-D-erythritol kinase. MDS, 2C-methyl-D-erythritol-2,4’-cyclodiphosphate synthase. HDS, 4-hydroxy-3-methylbut-2-enyl diphosphate synthase. HDR, 4-hydroxy-3-methylbut-2-enyl diphosphate reductase. For intermediates, GAP, D-glyceralfehyde-3-phosphate. DXP, 1-deoxy-D-xylulose 5-phosphate. MEP, 2C-methyl-D-erythritol 4- phosphate. CDP-cytidyl diphosphate. MEcDP, 2C-methyl D-erythritol-2,4-cyclodiphosphate. HMBDP-1-hydroxy-2-methyl- 2-(E)-butenyl-4-diphosphate. OP and OPP signify mono- and diphosphate moieties, respectively. Pi represents inorganic phosphate (**B**) Biosynthetic pathway of forskolin.

In plants, all terpenoids are formed by the condensation of five-carbon unit isopentenyl pyrophosphate (IPP) and dimethylallyl pyrophosphate (DMAPP) to form the precursor geranyl diphosphate (GPP), farnesyl diphosphate (FPP), and (*E, E, E*)-geranylgeranyl diphosphate (GGPP), finally form a terpenoid skeleton through the post-modification reaction. The five-carbon structure in plants can be obtained from the mevalonic acid (MVA) pathway in the cytoplasm and the 2-C-methyl-D-erythritol-4-phosphate (MEP) pathway in the plastid. The products of these two pathways can be interchanged through the plasma membrane. IPP and DMAPP can also undergo tautomerization under the action of isopentenyl pyrophosphate isomerase (*IDI*) (Fig. 3A). The MVA pathway and the MEP pathway have been extensively reported and will not be detailed in this review [79, 80].

After synthesizing IPP and DMAPP, geranylgeranyl pyrophosphate synthase (GGPPS) catalyzes the synthesis of GGPP, a precursor common to all diterpenoids, by three molecules of IPP and one molecule of DMAPP. GGPP could form the skeletons of various diterpenes under the action of different diterpene synthases. Then these skeletons are converted into different compounds through a series of enzymatic reactions, such as hydroxylation, peroxidation, methylation, glycosylation, and cleavage rearrangement. According to the functional domains of diterpene synthases, they can be divided into Class I, Class II, and Class I/ Class II diterpene synthases, Class I and Class II diterpene synthases contain DDXXD and DXDD domains, respectively. Class I/ Class II diterpene synthases contain both types of characteristic domains, having the characteristic of catalyzing two reactions at the same time. For example, abietadiene synthase isolated from *Gymnosperms* can catalyze GGPP to produce copalyl diphosphate (CPP), (+)-CPP, or *ent*-CPP, and then further be converted into abietadiene and *ent*-kaurene. Many related studies have been reported and will not be discussed here.

### Biosynthesis of Forskolin

Forskolin is a diterpenoid belonging to the labdane. It is produced uniquely to the root species of *C. forskohlii*, a genus of *Coleus* in the Labiaceae family. It mainly exists in the cork layer of the root of *C. forskohlii*, but the content is very low in plants. Forskolin can activate intracellular adenylyl cyclase to increase the concentration of cyclic adenosine monophosphates (cAMP). It has the effect of preventing platelet aggregation and antihypertension. It has been approved for treating asthma, glaucoma, hypertension, and other diseases.

**Fig. 4:**
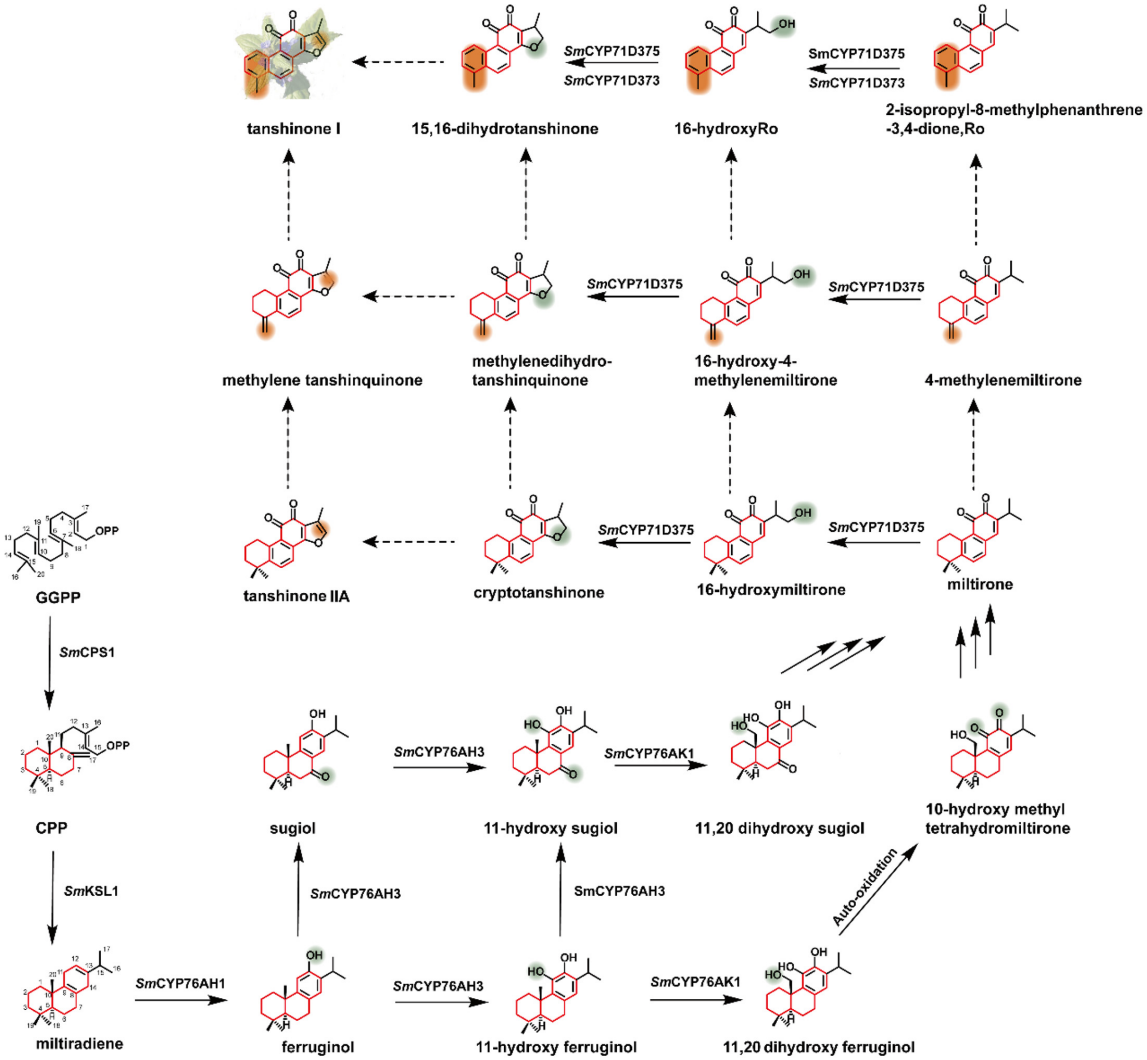
Biosynthetic pathway of tanshinones. Dash arrowhead indicates that the enzymes have not been identified.

In plants, GGPP is synthesized through the MEP pathway. The diterpene synthases are used to subsequently generate the basic diterpene skeleton, and finally, forskolin is obtained under a series of oxidases and acetyltransferases (Fig. 3B). Zerbe *et al*. performed transcriptome sequencing of *C. forskohlii* [81]. They identified six candidate diterpene synthases (*Cf*TPS1, *Cf*TPS2, *Cf*TPS3, *Cf*TPS4, *Cf*TPS14, and *Cf*TPS15) from the transcriptomic data. *Cf*TPS2 belongs to the Class II diterpene synthase. It can convert GGPP to form 8*α*-hydroxy-CPP and then cyclize into 13*R*‐manoyl oxide (13*R*-MO) by the Class I diterpene synthase *Cf*TPS3. However, in the presence of *Cf*TPS1 and *Cf*TPS3, or *Cf*TPS4, GGPP can be catalyzed to consecutively produce CPP and miltiradiene, a key hub in the biosynthesis of tricyclic diterpenes [82] (Fig. 4). In 2017, Pateraki *et al*. characterized the downstream enzymes in the forskolin biosynthetic pathway utilizing an agrobacterium-mediated transient expression system in tobacco. The results showed that *Cf*CYP76AH15, *Cf*CYP76AH8, and *Cf*CYP76AH17 could catalyze the formation of 11-oxo-13*R*-manoyl oxide from 13*R*-MO. *Cf*CYP76AH15 showed the highest activity and specificity among these three enzymes. *Cf*CYP76AH11 can convert 11-oxo-13*R*-manoyl oxide into 9-deoxy-7-deacetylforskolin, which is subsequently catalyzed to form 7-deacetylforskolin by *Cf*CYP76AH16. By co-expressing *Cf*DXS, *Cf*GGPPS, *Cf*TPS2, *Cf*TPS3, *Cf*CYP76AH15, *Cf*CYP76AH11, and *Cf*CYP76AH16, the intermediate of forskolin, 7-deacetylforskolin, was obtained. In addition, *Cf*ACT1-6 and *Cf*ACT1-8 were selected as candidate enzymes from ten acetyltransferases. It was found that *Cf*ACT1-8 has better specificity and could effectively convert 7‐deacetylforskolin into forskolin [83]. It was found that CFRB_1_ (*Alcaligenes faecalis*), a plant endophyte of *C. forskohlii*, could promote the synthesis of forskolin in plants by up-regulating the expression of the genes mentioned above [84].

### Biosynthesis of Tanshinones

Tanshinones are a class of lipid-soluble abietane found in *S. miltiorrhiza*, mainly distributed in the root and stem of *S. miltiorrhiza*. Up to now, more than 50 tanshinones have been isolated, including tanshinone I, tanshinone IIA, tanshinone IIB, dihydrotanshinone I, etc. Studies have proved that the phenanthrene-quinone structure of tanshinones is the basis of its cytotoxic effect, which can be combined with the DNA molecules to kill tumor cells [67].

In *S. miltiorrhiza*, GGPP is cyclized to miltiradiene under the diterpenoid synthases *Sm*CPS1 and *Sm*KSL1. Then various tanshinones are generated by different CYP450 enzymes, dehydrogenases, and demethylases (Fig. 4). Guo *et al*. identified six candidate CYP450s by analyzing the transcriptomic data of the root and the stem of *S. miltiorrhiza* [85]. They demonstrated that *Sm*CYP76AH1 could catalyze the generation of ferruginol from miltiradiene [86]. Guo *et al*. identified *Sm*CYP76AH3 and *Sm*CYP76AK1 that can sequentially catalyze multi-step oxidation reactions to ferruginol, generating two intermediates, 11,20-hydroxy ferruginol and 11,20-hydroxyl sugiol [87]. Mao *et al*. found that the sequence homology of *Sm*CYP76AH1 and *Sm*CYP76AH3 exceeded 80%, so they designed a series of *Sm*CYP76AH1 mutants to integrate the functions of the two enzymes based on homology modeling. Finally, CYP76AH1^D301E^ and CYP76AH1^V479F^ were certified to have the functions of both enzymes, and this strategy could improve the catalytic efficiency by reducing the transformation of intermediates [88]. Through the analysis and mining of genomic data, Ma *et al*. discovered the CYP71D subfamily in the genome of *S. miltiorrhiza*, and further studies demonstrated that CYP71D373 and CYP71D375 were involved in tanshinone biosynthesis [89].

There are many unknowns in the biosynthetic pathways of tanshinones and their intermediates. In recent years, many reports have paid more attention to the regulatory factors in the biosynthetic pathway. For example, Deng *et al*. found that *Sm*WRKY2 could increase the production of tanshinones by upregulating the expression of *Sm*DXS2 and *Sm*CPS [90]. *Sm*ERF73, a stress response transcription factor that belongs to the VII ethylene response factor family, plays a role in regulating gene expression related to tanshinones biosynthesis. When *Sm*ERF73 is overexpressed, seven genes involved in biosynthesis are up-regulated to increase the yield of tanshinones. Conversely, the transcript levels of these genes and the production of tanshinones will be down-regulated [91]. Besides, endophytic fungi can also enhance the synthesis of secondary metabolites in medical plants, *Penicillium steckii* DF33 can up-regulate the expression of key enzyme genes like *GGPPS*, *CPS1*, *KSL1* [92], the yield of tanshinone was increased, and the resistance of plants was also improved [93].

### Biosynthesis of Steviol Glycosides

STE-G are high-sweetness and zero-calorie sweeteners isolated from *S. rebaudiana* of the genus *Stevia* in the Asteraceae family. The synthesis of these compounds in plants occurs in chloroplasts. GGPP is also produced through the MEP pathway by using ^13^C-labeled glucose. GGPP is cyclized to *ent*-kaurene under the catalysis of class I terpene synthases, *ent*-copalyl diphosphate synthases (ent-CPPS), and class II terpene synthases, *ent*-kaurene synthases (ent-KS). The synthesized *ent*-kaurene is transported to the endoplasmic reticulum, where it is converted to *ent*-kaurenoic acid (KA) by the multifunctional CYP450 enzyme, *ent*-kaurene oxidase (KO).

**Fig. 5:**
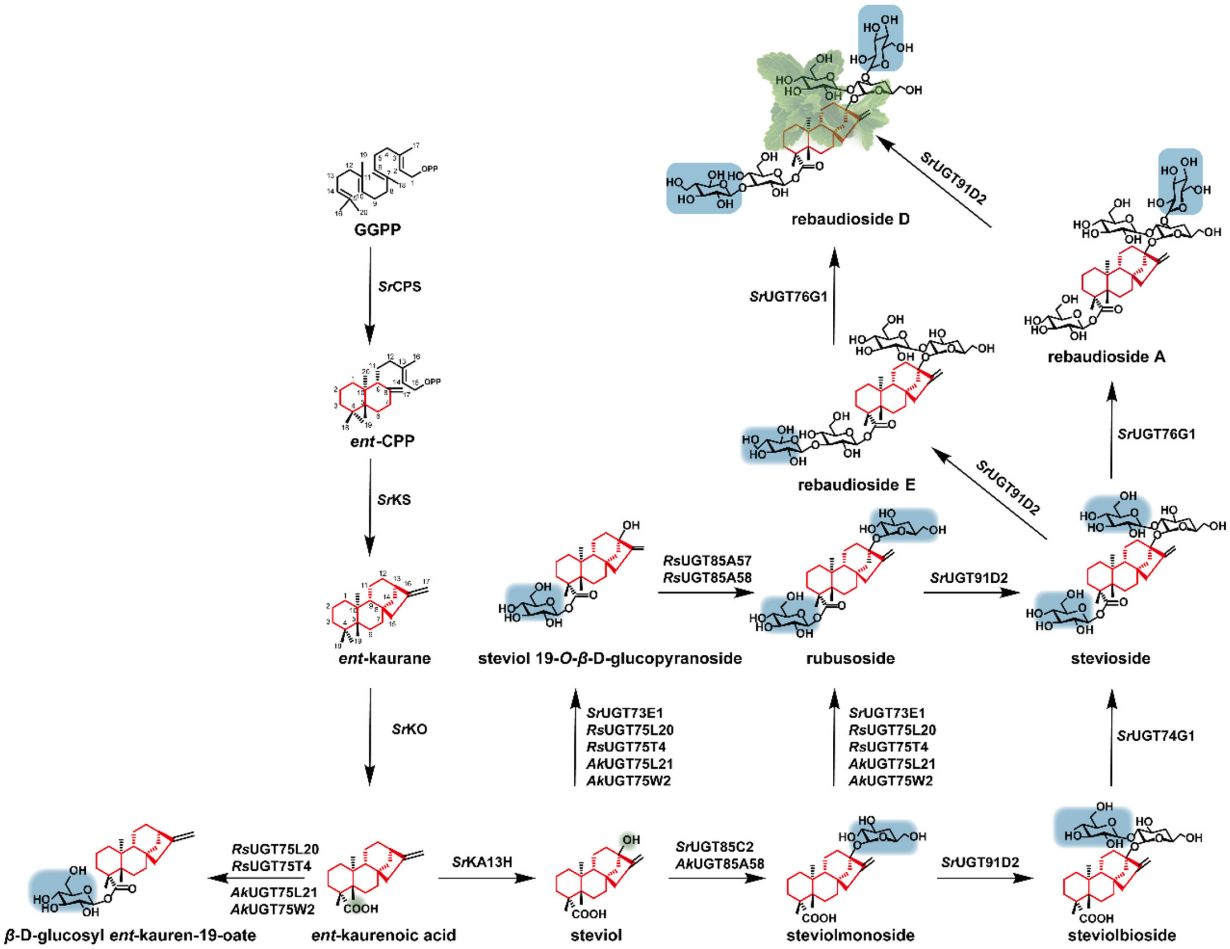
Biosynthetic pathway of steviol glycosides.

KA is converted to steviol by adding a hydroxyl at the C13 under the action of ent‐kaurenoic acid 13‐hydroxylase (KA13H). Steviol is the common precursor of all STE-G. Under the catalysis of glycosyltransferase *Sr*UGT73E1, one molecule of glucose is added to the carboxyl at C-19 of steviol to obtain steviol glycoside. Alternatively, under the coaction of *Sr*UGT85C2 and *Sr*UGT73E1, the glucosides at C-13 and C-19 are attached to form rubusoside. Then, stevioside can be achieved under the catalysis of glycosyltransferase *Sr*UGT91D2 that can form a 1,2-*β*-D-glycosidic bond on the glycosyl at C-13 of rubusoside. Moreover, with the catalyst of *Sr*UGT76G1 and *Sr*UGT91D2, stevioside is converted to rebaudioside A (Reb A) and rebaudioside E (Reb E), respectively. Rebaudioside D (Reb D) could be synthesized under the continuous catalysis of the above two enzymes [94](Fig. 5).

The most widely used STE-G on the market is Reb A, which has high sweetness and safety and has passed the safety certification of the United States Food and Drug Administration. However, Reb A has a bitter aftertaste. Compared to Reb A, Reb D and Reb M have one or two more glucose molecules attaching to the C-19 glycosyl, so the bitterness is significantly reduced. Wang *et al*. analyzed the transcriptomic data of *S. rebaudiana* and identified seven UDP-glycosyltransferases and 76 transcription factors that may be involved in the biosynthetic pathway of Reb D and Reb M [95]. Zhang *et al*. identified the glycosyltransferase *Os*UGT91C1 in rice. It can catalyze the conversion of Reb A to Reb D. Moreover, its F208M/F379A mutant reduced the formation of *β*-1,6 glycosidic bonds [96]. Dewitte *et al*. randomly mutated *Sr*UGT76G1 from *S. rebaudiana* and found that UGT76G1^T146G^ and UGT76G1^H155L^ could effectively reduce the production of by-products and increase the yield of Reb D or Reb M [97].

### Biosynthesis of Paclitaxel

**Fig. 6:**
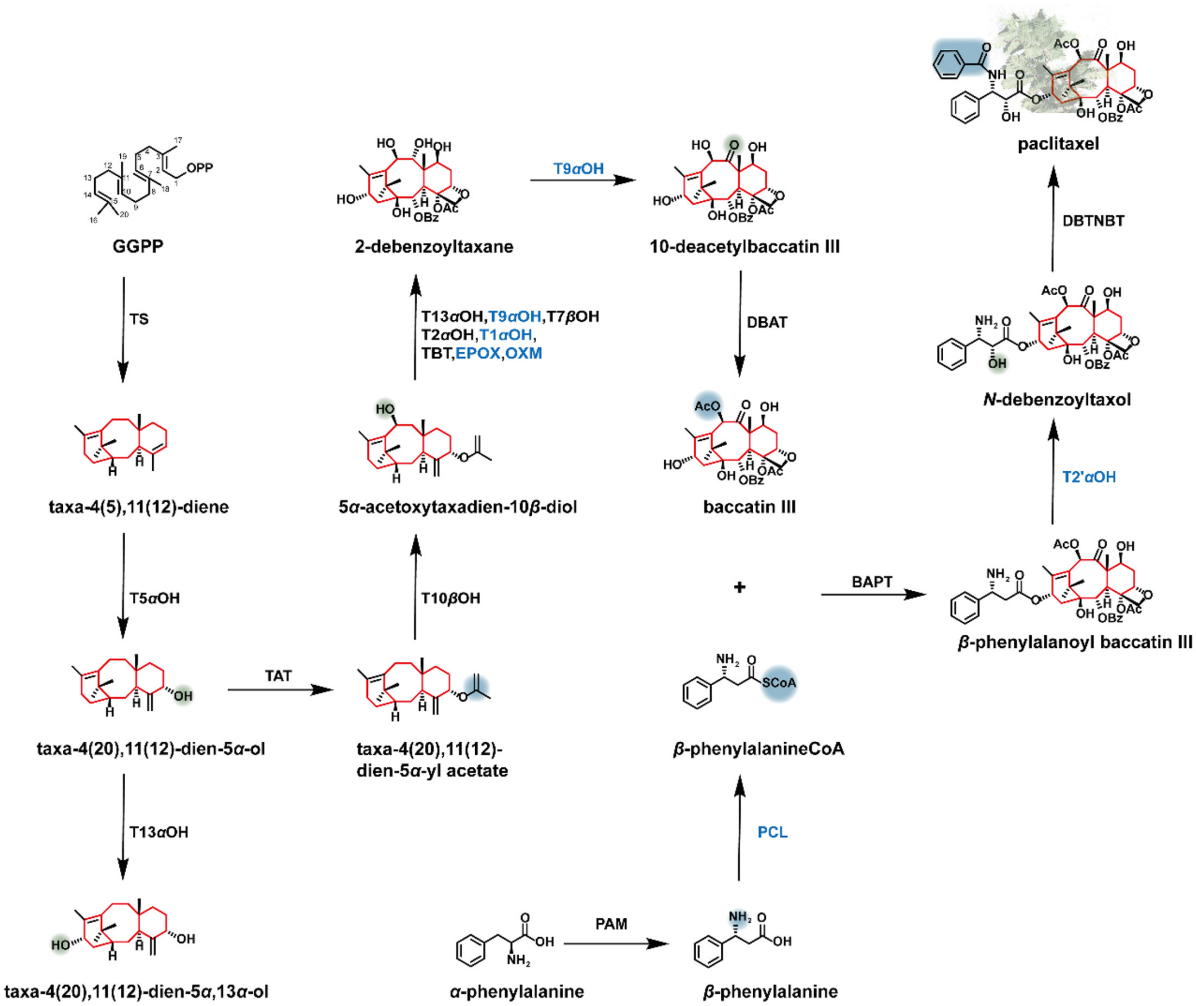
Biosynthetic pathway of paclitaxel. Blue font indicates that the enzymes have not been identified.

Paclitaxel is mainly isolated from the bark of *Taxus*. The demand for paclitaxel is increasing, but it is in short supply due to its low content in plants and the scarcity of the *Taxus* species. There is a consensus that constructing a cell factory to produce taxol by fermentation is the key to solve this problem. The biosynthetic process of paclitaxel has been divided into three parts: (1) biosynthesis of baccatin III, the parent nucleus of taxane, (2) biosynthesis of the phenylisoserine side chain, and (3) linkage of the side chain to the taxane skeleton (Fig. 6).[Table T1]

GGPP is first cyclized to taxadiene by the taxadiene synthase (TASY), a Class I diterpene synthase. Then taxadiene undergoes a series of hydroxylation at the C-1, C-2, C-4, C-5, C-7, C-9, C-10, and C-13 positions. The enzymes catalyzing hydroxylation at five positions of taxadiene have been successfully identified [98-100]. Stefan *et al*. found that taxadiene‐5α‐hydroxylase (T5αOH) can introduce a hydroxyl group at the C-5 position of taxadiene. The hydroxylation promotes the migration of double bond to form taxa‐4(20),11(12)‐dien‐5α‐ol [101]. Taxane 13*α*-hydroxylase (T13αOH) can convert taxane-5*α*-ol to taxadiene-5α, 13*α*-diol [102]. In addition, multi-step formylation and acetylation are involved in the formation of paclitaxel. The taxane-2*α*-ol-*O*-aectyltransferase (TAT) was identified from the *Taxus*, and it can catalyze the acylation of 5*α*-hydroxyl [103]. Walker *et al*. reported that taxane-2*α*-benzoyl transferase could convert the semi-synthetic substrate 2-debenzoyl-7,13-diacetylbaccatin III to form 7,13-diacetylbaccatin III. They also discovered that the 10-deacetylbaccatin III-10-*O*-acetyl transferase (DBAT) from *Taxus chinensis* could catalyze the reaction of 10-deacetylbaccatin III with acetyl-CoA to form bacatine III [104, 105] (Fig. 6).

Formation of the side chain of paclitaxel is the rate-limiting step in paclitaxel biosynthesis. With L-*α*-Phe as the precursor, *β*-Phe is obtained under the action of phenylalanine aminomutase (PAM) [106]. *β*-Phe is converted to *β*-Phe-CoA by *β*-phenylalanine-CoA ligase (PCL) [107]. The *β*-phenylpropanoyl-CoA is attached to the taxane scaffold at C-13 by taxoid C-13 *O*-phenylpropanoyltransferase (BAPT), the 2' position of the side chain is subsequently hydroxylated to form 3'-*N*-debenzoyltaxol by CYP450 hydroxylase [108]. Finally, the benzoylation occurs at the C3' position under the 3'-*N*-debenzoyl-2'-deoxytaxol-*N*-benzoyltransferase (DBTNBT) to complete the synthesis of paclitaxel [109] (Fig. 6). Zhang *et al*. identified a new C4*β*-C20 epoxidase and utilized it in conjunction with oxomutases / epoxidases, taxane 1*β*-hydroxylase, taxane 9*α*-hydroxylase, taxane 9*α*-dioxygenase, and phenylalanine-CoA ligase to successfully biosynthesize the key intermediate baccatin III and to convert baccatin III into paclitaxel in *Nicotiana benthamiana*, establishing a metabolic route to taxoid biosynthesis [110]. Through persistent efforts, the intricate biosynthetic pathways of taxanes have been gradually resolved.

By analyzing the functions of the enzymes identified so far, we can see that the hydroxylation and acylation on the parent nucleus are alternate, and the order of most reactions still needs to be determined. There are about eight CYP450 enzymes involved in paclitaxel biosynthesis, but only five have been identified. The corresponding CYP450 hydroxylases catalyzing reactions at the C-1, C-4, and C-9 positions are still unknown. Moreover, the enzymes involved in the biosynthesis of propylene oxide remain unknown. More efforts are needed to elucidate the complete paclitaxel biosynthetic pathway.

## Heterologous Production of Plant Diterpenoids

Using synthetic biology technology to produce active ingredients of medicinal plants in heterologous expression systems has great advantages, including short production cycles, independence from environmental factors, easy separation and purification, and easy to perform large-scale fermentation.

A variety of pharmaceutical ingredients have been produced de novo in chassis organisms. Keasling’s group has constructed a high-yield yeast engineering strain for the synthesis of the precursor of artemisinin, artemisinic acid. Then artemisinin can be obtained through a series of simple chemical reactions. The yield of artemisinic acid in a 100 m^3^ fermenter is equal to that of 8,000 acres of agricultural cultivation [111]. Chinese researchers have cooperated to construct a "Ginseng-Yeast" cell factory that can simultaneously produce oleanolic acid, protopanaxadiol, and protopanaxatriol, which enables the yeast to synthesize sapogenin [112]. L-homoserine is used as an important intermediate and additive in the industry. Zhang *et al*. achieved a yield of 11.1 g/l in *E. coli* through metabolic engineering [113].

### Heterologous Production in *E. coli*

*E. coli* has been a popular host in synthetic biology research because of its clear genetic background, rapid growth and metabolism, and genetic amenability. *E. coli* is often used as a chassis for producing terpenes. However, its IPP and DMAPP precursors cannot meet the needs of high-titer terpenoid production. Genetic modification is needed to enhance the metabolic flux in terpenoid biosynthesis, thus improving the yield of target products. Kong *et al*. overexpressed the 1-deoxyxylulose-5-phosphate synthase (*DXS*), farnesyl diphosphate synthase (*IspA*), and *IDI* in the MEP pathway to improve the titer of isoprenoid precursor. At the same time, they screened six GGPPSs from different species and eight *E. coli* hosts to determine the best combination for *ent*-kaurene production. Finally, *ent*-kaurene yield could reach 578 mg/l when expressing the GGPPS of *Rhodobacter sphaeroides* in *E. coli* MG1655 [114]. Using the strain optimization and precursor-enhancing strategies, Sun *et al*. selected *E. coli* K-12-derived strains with an enhanced MVA pathway as the host, followed by screening KO and adding protein fusion tags, the yield of KA could reach 250 mg/l. Subsequently, they used several methods, including N-terminal truncation, codon optimization, copy number increase, molecular docking, and site-directed mutagenesis, to improve the catalytic efficiency of oxidase KA13H. Finally, the titer of steviol reached 1.07 g/l [115].

Ajikumar *et al*. used multivariate-modular metabolic engineering in *E. coli* to partition the metabolic pathway of taxadiene into two modules: the upstream module of the endogenous MEP pathway for supplying IPP and the heterologous downstream module for terpene biosynthesis. By overexpressing four genes in the upstream module and two genes in the downstream module by changing the plasmid copy number and regulating the promoter strength, the yield of taxadiene reached 1.02 ± 0.08 g/l in the fed‐batch fermentations [9].

### Heterologous Production in Yeasts

Yeasts are also popular hosts for reconstituting the biosynthetic pathways of plant secondary metabolites. Many membrane-bound enzymes involved in plant metabolism, especially CYP450s, are relatively easier to be functionally expressed in yeast. *Saccharomyces cerevisiae* was the first yeast host for exogenous gene expression and reconstituting heterologous biosynthetic pathways. Dong *et al*. integrated *tHMG1* encoding the catalytic domain of 3-hydroxy-3-methylglutaryl coenzyme A reductase and *UPC2-1*, the G888D mutant of the transcription factor UPC2, into the chromosome of yeast BY4741 to improve the expression levels of *ERG13*, *ERG12*, and *ERG8* in the MVA pathway. In addition, they used the weak promotor P_HXT1_ to replace the native promotor of *ERG9* to reduce the flux to the squalene by-product, and the quantitative analysis showed that the content of GGOH reached 3.97 mg/l. Through site-directed mutagenesis to obtain mERG20, which can function as a GGPPS, then the fusion of ERG20 and mERG20 could enhance the titer of GGOH, which can be achieved at 12.54 mg/l [116].

Hu *et al*. used different approaches, such as overexpression of key enzymes in the MVA pathway, down-regulating the expression of genes in the competing metabolic pathways, and knocking down the transcription factors that repress terpenoids biosynthesis. Finally, the titer of GGPP reached 2.1 g/l. Then they used this strain to evaluate different miltiradiene synthases. It was found that the fusion protein composed of TPS1 from *C. forskohlii* and KSL1 from *S. miltiorrhiza* could produce 3.5 g/l of miltiradiene in a 5-L bioreactor [117]. The site-directed mutagenesis of CYP76AH15^A99I^ resulted in the enhanced production of 11-oxo-manoyl oxide, the intermediate of forskolin, by 5.6 folds [118]. Based on this, Ju *et al*. optimized the adaptations between *Cf*CYP76AHs, t66*Cf*CPR, and t30AaCYB5, and the yield of forskolin reached 759.42 μg/l. Moreover, multiple metabolic engineering strategies, including regulation of the gene copy numbers, amplification of the endoplasmic reticulum (ER) area, and enhancement of cofactor metabolism, were implemented to improve the metabolic flux of 13*R*-MO during forskolin generation. The yield of forskolin reached 21.47 mg/l in the shake flask and 79.33 mg/l in a 5-L fermenter [119].

Wei *et al*. improved carnosic acid biosynthesis efficiency in yeast through the co-expression of CYP450s and CPR enzymes, generating fusion protein, utilizing endoplasmic reticulum engineering, and increasing the supply of cofactors to improve the carnosic acid yield to 24.65 mg/l in shake flasks [120]. Xu *et al*. remodeled the complex metabolic networks of rubusoside by modular engineering. By overexpressing the rate-limiting enzymes and using the FPS^F112A^ mutant to reduce the generation of shunt products, they increased the yield of *ent*-kaurene by 33.9 times. In addition, the combination of different strategies, including regulating the cellular stress response, activating the efflux pump, and knocking out bypass pathways, resulted in the production of 1368.6 mg/L rubusoside in a 15-L bioreactor [94].

Kildegaard *et al*. used the oleaginous yeast *Y. lipolytica* to construct the biosynthetic pathway for GAs. The production of KA, the GA precursor, reached 3.75 mg/l by optimizing the MVA pathway and overexpressing the rate-limiting enzymes in the pathway. Then they generated a GA4-producing strain by introducing the enzymes involved in GAs biosynthesis, resulting in the production of 17.29 mg/l GA4 [38].

### Other Chassis Cells

In addition to *E. coli* and yeast, other microorganisms were also used as chassis for diterpenoids biosynthesis. Green microalgae can convert CO_2_ into valuable products. The chloroplasts of the photoautotrophic green microalgae (*C. reinhardtii*) contain large amounts of GGPP. Therefore, it holds tremendous potential as an efficient and sustainable heterologous chassis for the biosynthesis of plant diterpenoids. By combining synthetic biology and metabolic engineering strategies, Einhaus *et al*. constructed an efficient and stable microalgae chassis to produce sclareol. Then they evaluated and fused GGPP synthase and sclareol synthase from different sources. The yield of sclareol reached 656 ± 9.5 mg/l by optimizing the fermentation conditions [121].

*Streptomyces* is an important source of natural products. With the development of genome sequencing technology, a variety of terpene synthases have been discovered in *Streptomyces*. Therefore, *Streptomyces* are also good chassis to produce diterpenes. Khalid *et al*. constructed a terpenoid-biosynthetic platform by enhancing precursor supply and screening promoters in *Streptomyces reveromyceticus* SN-593 [122]. *Streptomyces avermitilis* SUKA22 is a model strain for gene expression. Yamada *et al*. expressed seven different diterpene synthases silencing in the original strains and produced 11 new terpenoids, among them, odyverdienes A and B each displaying a novel diterpene skeleton [123].

## Summary and Scope

With their amazing structural diversity and broad biological activities, natural products have provided many pharmaceutical agents and intermediates for human beings. Terpenoids are a large group of natural products, among which diterpenoids are rich in structure and have attracted human attention because of their extensive biological activities like antineoplastic activities, anti-inflammatory activities, antibacterial abilities, and many other strong functions. However, their complex and varied structures pose challenges in terms of separation and purification. With the increased demand for bioactive diterpenes, extracting diterpenes from plants is far from satisfying human needs. By analyzing the biosynthetic pathways for plant diterpenes, the yield of diterpenoids can be effectively improved by rational construction and optimization of the biosynthetic pathway in heterologous hosts. The biosynthetic pathways of several diterpenes have been completely or partially resolved in recent years. However, due to the complex structures of diterpenes and the discontinuity of gene clusters in plants, it is a great challenge to elucidate the diterpene biosynthetic pathways. The development of next-generation sequencing techniques and bioinformatic analysis can effectively reduce the candidate genes involved in the biosynthesis of plant diterpenoids. By screening candidate genes in well-established chassis, such as *E. coli* and yeast, the yield of diterpenoids in the heterologous hosts can be improved efficiently.

Significant progress has been made in the production of diterpenoids in microorganisms recently. Representative studies such as: through the pathway mining, analyzing and assembling the biosynthesis of steviol glycosides, the heterologous synthesis of plant-derived steviol glycosides was realized in *E. coli* chassis [124]. The natural fragrance sclareol is the raw material of ambergris, whose efficient synthesis was realized through the analysis of its synthesis pathway, reconstruction and optimization in *S. cerevisiae*, and the global regulation of central metabolic pathway [125]. The field holds immense promise and is of paramount importance for resource development, medicine, and the chemical industry. In early studies, characterizing and expressing key enzymes involved in biosynthesis were an important strategy to increase the yields of diterpenoids. However, the yield improvement was often far from satisfactory. More and more research focus on improving the catalytic efficiency of enzymes by site-directed evolution and truncation or fusion of enzymes to improve the substrate delivery efficiency. In the heterologous hosts, knocking out the competitive pathways or suppressor genes or knocking down their expression by replacing the native promoters with weaker promoters can also reduce the production of by-products. Some diterpenes are cytotoxic, inhibiting the growth of the host, and limiting the accumulation of products. Therefore, enhancing the efflux of compounds and the tolerance of strains are also helpful in improving the yield of compounds. In addition, introducing key genes in the biosynthetic pathways into different chassis may also generate new products, thereby enriching the types of diterpenoids and contributing to the discovery and development of new diterpenes.

In conclusion, obtaining genes related to the diterpenoid biosynthetic pathway from plant genomes is the basis for producing active components utilizing synthetic biology. The metabolic pathway of engineering bacteria was redesigned and constructed using synthetic biology technology, and functional gene elements were assembled into chassis organisms to build efficient cell factories. As research on diterpene components deepens, the demand for diterpenes in medical and natural environment outpaces supply. This comprehensive strategy provides ideas and methods not only for the biosynthesis of diterpenoids but also for other kinds of natural products. With the unlocking of the bottleneck and limiting factors in the biosynthesis of diterpenes, the production and utilization of diterpenoids will also make more significant progress.

## Figures and Tables

**Table 1 T1:** Overview of production of plant diterpenoids in different hosts.

Host	Diterpene	Metabolic engineering strategies	Titer (mg/l)	Reference
*E. coli*	*ent*-kaurane	◆ Screening of optimal GGPPS and *E. coli* host ◆ Overexpression of three key enzymes in MEP pathway ◆ Culturing strain in a bioreactor	578	[114]
*E. coli*	Steviol	◆ Optimization of the upstream pathway ◆ Screening of proper *E. coli* host and kaurene oxidase ◆ Truncating the N terminus-modified mutants of kaurene oxidase and attachment of soluble tags ◆ Codon optimization and increasing the copy number of *CPR* ◆ Introduction of cytochrome b5 (CYB5) ◆ Site-directed mutation of *At*CYP714A2	1073.8	[115]
*E. coli*	Taxadiene	◆ Modularization of the taxadiene metabolic pathway ◆ Using systematic multivariate analysis to achieve a balance of the two modules	1020	[9]
*S. cerevisiae*	Miltiradiene	◆ Overexpression of the pathway genes ◆ Downregulation of *Erg9* ◆ Knocked out transcriptional regulators ◆ Optimization of the medium ◆ Fusion of *Cf*TPS1 and *Sm*KSL1 ◆ Protein modification of *Sm*KSL1	3500	[117]
*S. cerevisiae*	Forskolin	◆ Fusion of *BTS1* and *ERG20*^F96C^ ◆ Overexpression of *HMG1* ◆ Truncating the N terminus of *Cf*CPR ◆ Fusion of *Cf*CYP76AHs and tR~tB ◆ Regulation of copy numbers of the target genes, amplification of the endoplasmic reticulum (ER) area and cofactor metabolism enhancement ◆ Fed-batch fermentation	21.47	[119]
*S. cerevisiae*	Carnosic acid	◆ Overexpression of *BTS1*-GGGS-*ERG20*^F96C^p ◆ Codon-optimization, N-terminus truncation, and fusion of *tSm*CPS and *tSm*KSL ◆ Used *Sm*CPR from *S. miltiorrhiza* ◆ Co-expression of *Sm*CPR~*t28Sp*Cytb5 fusion protein and CYP76AH1 ◆ Overexpression of *Sc*CTA1 and *Sc*CTT1 ◆ Overexpression of *INO2*, the *HEM3* (heme synthase) gene, and the NADH kinase gene (*POS5*) ◆ Batch and fed-batch fermentation	24.65	[120]
*S. cerevisiae*	Rubusoside	◆ KS from *G. fujikuroi*, KO, CPR1, UGT74G1, and UGT85C2 from *S. rebaudiana*, KAH from *Arabidopsis thaliana* ◆ Overexpression of *tHMG1* and *IDI1* ◆ Site-directed mutation of *FPS*^F112A^ ◆ Replacing promoter of *INO2* with a stronger one ◆ Overexpression of the efflux-pump *PDR11* and the stress-response factor *MSN4* ◆ Knocking out *GAL7* and overexpression of *PGM2*	1368.6	[94]
*Y. lipolytica*	Gibberellin	◆ Downregulating the endogenous squalene synthase gene ◆ Choosing *At*CPS, *At*KS, *At*KO, *At*ATR2 ang *Yl*Cyb5 to producing *ent*-KA ◆ Codon-optimization of all genes ◆ Gene *At*C20ox and *At*C3ox were expressed under the control of the strong promotors *Pr*Exp and *Pr*Tefintron, respectively ◆ N-terminus truncation of *At*CPS, *At*KS, and *At*KO and fusion of CPS and KS	GA4 17.29 GA3 2.93	[38]
*C. reinhardtii*	Sclareol	◆ Codon optimization of terpene synthase ◆ All transgenes were driven by the PSAD promoter and FDX1 terminator ◆ Using GGPPS from *C. reinhardtii* and sclareol synthase from *S. sclarea*	656	[121]
